# Depressive Symptoms, Lack of Physical Activity, and Their Combination Towards Health Care Utilisation Frequency

**DOI:** 10.3390/ijerph16234697

**Published:** 2019-11-26

**Authors:** Sandra Haider, Igor Grabovac, Anita Rieder, Thomas Ernst Dorner

**Affiliations:** Department of Social and Preventive Medicine, Center for Public Health, Medical University of Vienna, 1090 Vienna, Austria; sandra.a.haider@meduniwien.ac.at (S.H.); anita.rieder@meduniwien.ac.at (A.R.); thomas.dorner@meduniwien.ac.at (T.E.D.)

**Keywords:** depressive symptoms, physical activity, health care utilisation

## Abstract

Depressive symptoms and lack of physical activity are independent factors that lead to higher health care utilisation, often occurring simultaneously. We aimed to assess the effects of depressive symptoms, lack of aerobic physical activity (PA), and the combination of those factors on the probability of using in- and outpatient health care services in men and women. Data from 15,770 people from the nationally representative Austrian Health Interview Survey (AT-HIS) were used. In analysis, depressive symptoms, adjusted for sociodemographic, health related, and lifestyle-related factors were associated with higher odds of outpatient health care utilisation (OR: 1.60; 95% CI: 1.19–2.14) in men and (OR: 2.10; 95%CI: 1.65–2.66) in women, and with higher odds of inpatient health care utilisation (OR: 1.52; 95% CI: 1.09–2.10) in men and (OR: 2.09; 95% CI: 1.64–2.68) in women. However, depressive symptoms were not associated with higher health care utilisation in the fully adjusted models. In men, co-existence of depressive symptoms and lack of health enhancing physical activity (HEPA) was associated with higher odds of using inpatient health care services, compared to the presence of only one or none of the factors. In conclusion, our results show that depressive symptoms are associated with more health care utilisation in both men and women and that the co-existence of both depressive symptoms and lack of HEPA elevated the odds for inpatient health care utilisation in men even more.

## 1. Introduction

Depression is a major public health problem, estimated to affect more than 300 million people globally [1,2]. In Austria, the prevalence of depression in adults in 2014 was reported at 10% for women and 6% for men [3], which puts the depression burden in Austria higher in comparison to both other European countries and worldwide [4,5].

Depression and depressive symptoms are associated with an increase in health care utilisation (primary and secondary level of care, medication use, laboratory analyses), creating added pressure on the social and health care systems [6,7,8]. Although no exact cost calculations are made for Austria, these are expected to be very high as approximately 10% of the entire population received treatment for psychological issues in 2009 [9]. A study on the impact of psychological distress and primary and secondary level of care utilisation in Austria reported that psychological distress was associated with higher odds of visiting both general practitioners (GPs) and medical specialists within the last four weeks [10]. Moreover, between 1995 and 2015, disability pensions based on mental health issues have tripled, and in 2017, mental health issues had the second highest average length of sick leave [11]. These data are worrying, as these reports are possibly underestimations given monitoring issues in Austria (no specialist registries), as well as misdiagnoses, mental health illness stigma, and other issues [12].

Another important health hazard is lack of physical activity (PA), which has been associated with poorer physical and mental health outcomes and with higher health care utilisation, as people who report lack of PA and have more sedentary behaviour also tend to use health care service more commonly than active individuals [13]. In fact, PA is one of the most important tools in health promotion, prevention, and curation of various diseases as well as health maintenance and, as such, may reduce health care utilisation [14]. Such PA consists of leisure time physical activity (LTPA), occupational PA, and physical activity during transportation [15]. According to Finger and colleagues, LTPA and PA during transportation with the bicycle may be considered as a health enhancing physical activity (HEPA) [16]. In Austria, about half of the adult population do not fulfil the minimal requirements for aerobic HEPA, and about two-thirds do not reach the recommended two times a week muscle strengthening HEPA. Furthermore, it was reported that participants who did not fulfil aerobic HEPA recommendations had higher odds of visiting their GP [17].

Although depressive symptoms and lack of physical activity are independent factors that lead to a higher health care utilisation, they often occur simultaneously. People with depressive symptoms often have lack of motivation and, therefore, often do not participate in adequate HEPA, and vice versa, people with low HEPA levels, have a higher risk for developing depressive symptoms and depressive disorders [18,19,20]. As a domain of PA, exercise appears to be as effective as psychological and pharmacological therapies, while at the same time reducing the risk of other common chronic diseases [21,22,23,24].

Because of the associations between depressive symptoms and lack of HEPA, not only the association of the individual factors with health care utilisation is of interest, but also the combination of those factors. Thus, the aim of the paper was to assess the effects of depressive symptoms, lack of HEPA, and the combination of those factors on the probability of using in- and outpatient health care services.

## 2. Materials and Methods

We used the data from the Austrian Health Interview Survey (AT-HIS) from 2014. The AT-HIS is a cross-sectional study, which was conducted from October 2013 to June 2015 mainly via computer-assisted telephone interviewing. However, questions concerning physical activity were asked via paper questionnaire. Overall, 38,768 people were invited to participate and, finally, a total of 15,771 people were included [3]. For the present analysis, we included all men and women older than 15 years. This secondary analysis was approved by the research ethics committee of the Medical University Vienna (EK# 2211/2015).

### 2.1. Measurements

*Depressive symptoms* were assessed using the “Personal Health Questionnaire Depression Scale” (PHQ-8) [3,25]. This questionnaire includes eight different questions and ranges from 0 (not depressive) to 24 points. In the present analysis, people with a score >10 points were defined to have depressive symptoms [25].

*Aerobic health enhancing physical activity (HEPA)* was assessed with the domain “aerobic LTPA” with the “European Health Interview Survey-Physical Activity Questionnaire” (EHIS-PAQ) [3,16] with the following two questions:

“In a typical week, on how many days do you carry out sports, fitness or recreational (leisure) activities for at least 10 minutes continuously?” and

“How much time in total do you spend on sports, fitness or recreational (leisure) physical activities in a typical week?”.

To calculate the HEPA, we have added up the minutes per week spent cycling, as recommended by Fingers and colleagues [16].

If people, (1) did aerobic HEPA <150 min a week, these people were considered to have a “lack of HEPA”; otherwise, they were considered as “physical active”.

*Health care utilisation* was assessed for both outpatient and inpatient care.

*Outpatient health care utilisation* included visits to a (1) GP, (2) medical specialist, (3) hospital outpatient clinic or emergency room in the past 4 weeks.

*Inpatient care utilisation is* based on a question “Have you spent one or more nights in a hospital in the past year?”.

*Sociodemographic parameters* (age, gender, educational level, partnership status) and *health information* (chronic illnesses and health problems, smoking status, alcohol intake, BMI) were included in the analysis.

*Education level* was assessed with the question “What is your highest school degree?”. The response options “compulsory school” and “vocational school” were converted to “primary education”; “commercial school”, “apprenticeship”, and “high school certification “were summarised to “secondary education”; “university” or “university of applied sciences” and “other degrees after high school” were summarised to “tertiary education”.

*Partnership* status was assessed with the question “What is your current marital status?”. The response options “single”, “widowed”, “divorced”, and “no answer” were summarised to “living alone”. “Married, registered partnership, living together”, “married, registered partnership living separate” were summarised to “living in a partnership”.

*Chronic illnesses and health problems* were assessed with the question “Did you have one of the following illnesses or health problems in the last 12 months?”. A list of 16 somatic illnesses was read out to the participants.

*Smoking status* was assessed with the question “Are you smoking?”. The response options “every day” and “seldom” were summarised to “currently smoker”. Otherwise, the parameter value was “currently non-smoker”.

*Alcohol intake* was assessed with the question “How often have you drank alcohol in the last 12 months?” The response options “daily, nearly daily”, “5–6 days a week”, “3–4 days a week” and “1–2 days a week” were summarised to “>once a week”. The response options “2–3 days a month”, “once a month”, “<once a month”, “nothing in the last 12 months”, “never or only some swallow in my life” were summarised to “<once a week”.

*Body mass index* was calculated with the self-reported body high and body weight. All participants were categorised into underweight (<18.5 kg/m^2^), normal weight (18.5–24.9 kg/m^2^), overweight (25.0–29.9 kg/m^2^), and obese (≥30.0 kg/m^2^) [26].

### 2.2. Statistical Analysis

Descriptive analyses are presented as percentages. Binary logistic models using inpatient and outpatient health care utilisation (yes, no) as dependent variables was performed as crude and adjusted models. Model 1 was the unadjusted model. Model 2 was adjusted for age, education level, and partnership status. Model 3 was additionally adjusted for each of the assessed 16 chronic somatic illnesses, smoking status, alcohol intake, and body mass index. Model 4 was additionally adjusted for depressive symptoms in the analysis with lack of HEPA as a dependent variable, and for lack of HEPA in the analysis with depressive symptoms as a dependent variable. All these analyses were stratified for sex.

Combinations of depressive symptoms and lack of HEPA in relation to health care utilisations are shown as synergy index, population attributable fraction and relative excess risk due to interaction [27,28,29]. The synergy index is the ratio of observed effect (combined exposure of risk factors) to the expected effect. The population attributable factor indicates the proportion of health care utilisation that would theoretically be avoided if the interaction of the two exposures was eliminated. The relative excess risk due to interaction is a metric due to departure from the additivity of effects. For the calculation of the confidence intervals, the delta methods, a straightforward Taylor expansion of the variances and covariances were used [27].

## 3. Results

In total, 15,770 people (48.6% male) were included (Table 1). The majority of the people were between 30 and 64 years old, had finished secondary school, and lived in a partnership. Seventy percent were non-smokers, and about half of the participants drank alcohol more than once a week. The most common chronic illnesses were chronic back pain with one-fourth of the subjects being affected, followed by allergies and high blood pressure. Heart attack, stroke, liver cirrhosis was prevalent in less than 1% of the sample. Half of the participants were normal weighted, and one-third were overweight. Taking a look at the health care utilisation in the last four weeks, two-thirds used the outpatient health care system and less than 15% of the inpatient health care system. Half of the included participants performed HEPA for at least 150 min, thus achieving the HEPA recommendations for aerobic HEPA. Based on the PHQ scores, under 5% had depressive symptoms.

The results of the logistic regression (Table 2 and Table 3) showed that men and women with depressive symptoms had an almost three times higher odds for outpatient or inpatient health care utilisation in the unadjusted model when compared to those without depressive symptoms. These associations remained significant in the fully adjusted model, where men had 60% and 52% higher odds for using the out- or inpatient health care system, respectively. In the fully adjusted model, women had an even more than double chance for out- and inpatient health care utilisation.

In women, a lack of HEPA was associated with 17% higher odds for outpatient health care utilisation, and 32%higher odds for inpatient health care utilisation in the unadjusted model. This association was not significant in the fully adjusted model. In men, however, there was no significant association between outpatient or inpatient health care utilisation and lack of HEPA in the unadjusted model, but in the fully adjusted model, men with lack of HEPA had a significantly lower chance of outpatient health care utilisation.

In Table 4, the results of men and women with the possible combinations of lack of HEPA and depressive symptoms towards outpatient health care utilisation are shown. Sixty-six percent of men and 73% of women with both, lack of HEPA and depressive symptoms used the outpatient health care system, compared with only 40% of men and 46% of women with sufficient HEPA and no depressive symptoms. In the fully adjusted model, men and women with both conditions had 1.43 and 2.02 higher odds for outpatient health care utilisation compared to men and women with neither of those conditions, respectively. Odds were even higher in men and women with sufficient HEPA and depressive symptoms than in those with lack of HEPA and depressive symptoms. There was no significant interaction effect between lack of HEPA and depressive symptoms towards outpatient health care utilisation.

In Table 5, the results of men and women with the possible combinations of lack of HEPA and depressive symptoms towards inpatient health care utilisation are shown. A total of 33% of men and 29% women with both lack of HEPA and depressive symptoms used the inpatient health care system, compared with only 14% of men and 13% of women with sufficient HEPA and no depressive symptoms. In the fully adjusted model, men and women with both conditions had 1.52 and 1.75 higher odds for inpatient health care utilisation compared to men and women with neither of those conditions, respectively. In women, odds for those with sufficient HEPA and depressive symptoms towards inpatient health care utilisation were largely higher compared to women with lack of HEPA and depressive symptoms. This was, however, not the case for men. The synergy index of the combination of lack of HEPA and depressive symptoms towards inpatient health care utilisation was significantly <1 for men in the unadjusted model and for women in the adjusted models, meaning a certain neutralisation of both factors towards the outcome.

## 4. Discussion

The results of this nationally representative Austrian data showed that depressive symptoms are a factor promoting the utilisation of both the outpatient and the inpatient health care system. Additionally, lack of HEPA was a factor promoting health care utilisation only, however, in women and only in the unadjusted models. When compared to the presence of depressive symptoms, lack of HEPA played a minor role. Furthermore, a co-existence of depressive symptoms and lack of HEPA was associated with higher odds of using inpatient services when compared to the presence of one or none of the two factors in men.

The clear association between depressive symptoms and frequency of health care utilisation is in line with previously published studies [6,7,8]. We saw this association in the utilisation of both outpatient and inpatient health care services and in men and women, whereby women had higher odds of health care utilisation compared to men. As sex has been identified as a predisposing factor in care seeking [30,31], we hypothesise two possible reasons for this difference. On the one hand, studies indicate that rates of undiagnosed depression are substantially higher among men and that men seek help from mental health services less often than women and are less likely to be diagnosed [32,33,34,35]. On the other hand, studies indicate gender differences in self-reporting of depressive symptoms. For example, studies reported that women more often report somatic symptoms associated with depression (fatigue, pain, loss of appetite) when compared to men [36,37,38].

Our analysis also showed that lack of HEPA was not clearly associated with higher utilisation of out- or inpatient health care utilisation. Contrary to this, in men, in the fully adjusted model, lack of HEPA was significantly associated with a lower chance of outpatient healthcare utilisation. In women, having sufficient HEPA plus being affected by depressive symptoms was associated with a remarkable higher chance for inpatient healthcare utilisation compared to having lack of HEPA plus being affected by depressive symptoms. Reasons for this may lie in the cross-sectional nature of the study, and we can only hypothesise about the reason for this finding. It may be, that in men with chronic diseases, regular HEPA is part of the treatment regime of the chronic condition, and the higher health care consultation with more HEPA is also an expression of the chronic condition. Similarly, it may be that in women with depressive symptoms, especially when more severe, HEPA is part of the clinical treatment, and due to the higher severity, those with depressive symptoms and sufficient HEPA have a higher inpatient health care utilisation compared to those with depressive symptoms and lack of HEPA.

However, our analysis did show higher inpatient health care utilisation in men with both, depressive symptoms and lack of HEPA when compared to those with only one or none of the two factors. Research suggests that people with depression show more sedentary behaviour and report less HEPA and that a lack of HEPA was identified as a risk factor for developing depression [20]. Therefore, it may be that, in men at least, those with depressive symptoms and lack of HEPA are the more severe cases, and this leads to a higher chance of inpatient health care utilisation. Furthermore, international guidelines often indicate the need to motivate patients with depression in HEPA [39,40]. HEPA has demonstrated to reduce the symptoms of depression [41] via immunological modification and effects the endorphin and monoamine secretion [42,43]. Meta-analyses have shown that HEPA protects from the onset and reoccurrence of depressive symptoms and improves the quality of life of patients with depression [21]. Moreover, various studies have indicated that HEPA has a positive effect on mental well-being. They also note that the effect of HEPA on depressive symptoms is as effective as psychological and pharmacological treatment and reduces the risks of other common chronic diseases [44]. Variability of potential PA interventions is wide as any type of PA can have beneficial effects on depressive symptoms [45].

Strengths of the present analysis are the large and nationally representative sample. However, the results need to be viewed in light of the study limitations. Given the self-reported nature of the questionnaire, it is possible that reporting bias led to some data distortion. It should especially be mentioned that physical activity was assessed with a questionnaire. Although the validity and reliability of the EHIS-PAQ were assessed and deemed acceptable [46], physical activity questionnaire showed limited reliability [47]. Additionally, it has to be taken into account that we did the calculations with HEPA, not including the time spend with occupational PA. We decided to exclude occupational PA, as studies have shown that occupational PA does not necessarily promote health [48,49,50,51]. Finally, the cross-sectional nature of the study design prevents drawing causal and chronological conclusions.

## 5. Conclusions

In conclusion, our results of nationally representative Austrian data showed that depressive symptoms were associated with more health care utilisation in men and women. The co-existence of both depressive symptoms and lack of HEPA was associated with higher odds for inpatient health care utilisation in men compared to the existence of only one or none of the conditions. Given the increasing prevalence rates of depression globally and simultaneous low levels of HEPA, this analysis further suggests the importance of public health interventions aiming at increasing HEPA as these may lower the health care utilisation and, thus, lower the ever-growing pressures on the social and health care systems. Further studies should focus on how these different factors intersect and influence health outcomes.

## Figures and Tables

**Table 1:** Descriptive statistic of all included participants (all numbers in %).

	Total *N* = 15,770	Men *N* = 7669	Women *N* = 8100
**Age**			
15–29 years	21.5	22.5	20.6
30–64 years	57.5	58.8	56.3
65+ years	21.0	18.8	23.1
**Education level**			
Primary	22.3	17.2	27.0
Secondary	48.4	53.7	43.5
Tertiary	29.3	29.0	29.5
**Partnership status**			
Living alone (single, widowed, divorced)	34.6	29.9	39.1
Living in a partnership	65.4	70.1	60.9
**Smoking status**			
Currently smoker (yes)	30.0	32.9	27.2
Currently non-smoker (yes)	70.0	67.1	72.8
**Alcohol**			
<once a week	43.5	30.4	55.9
>once a week	56.5	69.6	44.1
**Chronic somatic illnesses and health problems**			
Bronchial asthma, (yes)	4.4	4.0	4.7
Chronic Bronchitis, COPD, Emphysema, (yes)	4.2	4.0	4.4
Heart attack, (yes)	1.0	1.4	0.6
Coronary heart disease, (yes)	2.2	2.2	2.2
Blood pressure, (yes)	21.1	20.5	21.7
Stroke, (yes)	0.8	0.8	0.8
Arthritis, (yes)	12.0	8.3	15.4
Chronic back pain, (yes)	24.4	22.9	25.8
Chronic neck pain, (yes)	18.5	14.1	22.7
Diabetes mellitus type 2, (yes)	4.9	5.4	4.5
Allergies, (yes)	24.2	21.8	26.5
Liver cirrhosis, (yes)	0.2	0.2	0.2
Urinary incontinence, (yes)	3.6	2.0	5.2
Chronic kidney problems or kidney failure, (yes)	1.5	1.1	1.8
Chronic headaches, (yes)	6.7	3.9	9.4
Stomach or intestinal ulcer, (yes)	2.5	2.3	2.7
**Body mass Index (kg/m^2^)**			
Underweight (<18.5 kg/m^2^)	2.8	1.2	4.3
Normal weight (18.5–<24.9 kg/m^2^)	50.4	43.8	56.6
Overweight (25.0–<29.9 kg/m^2^)	32.5	39.4	25.9
Obesity (≥30 kg/m^2^)	14.3	15.6	13.2
**Outpatient health care utilisation in last 4 weeks**			
General practitioner, (yes)	30.8	28.7	32.8
Medical specialist, (yes)	21.7	18.5	24.6
Hospital outpatient clinic or emergency room, (yes)	10.8	10.6	11.0
**Inpatient health care utilisation in last 12 months**			
Hospital (yes)	14.8	14.3	15.2
**Health enhancing physical activity**			
aerobic exercise ≥150 min per week	50.1	53.1	47.2
aerobic exercise <150 min per week	49.9	46.9	52.8
**Depressive symptoms**			
0–9 points on the Personal Health Questionnaire Depression Scale	95.7	96.6	94.9
10–24 points on the Personal Health Questionnaire Depression Scale	4.3	3.4	5.1

Results are given in percentages.

**Table 2:** Crude and adjusted ORs for outpatient health care utilisation related to physical inactivity and depressive symptoms.

Outpatient Health Care Utilisation					
	All *N* (%)	Model 1 (Unadjusted) OR (95% CI)	Model 2 OR (95% CI)	Model 3 OR (95% CI)	Model 4 OR (95% CI)
**Men**					
Lack of health enhancing physical activity	3598 (46.9)	1.03 (0.94–1.13)	0.95 (0.86–1.04)	0.89 (0.81–0.98)	0.89 (0.80–0.98)
Depressive symptoms	260 (3.4)	2.89 (2.23–3.75)	2.54 (1.95–3.31)	1.58 (1.18–2.12)	1.60 (1.19–2.14)
**Women**					
Lack of health enhancing physical activity	4276 (52.8)	1.17 (1.07–1.28)	1.14 (1.04–1.28)	1.06 (0.97–1.16)	1.05 (0.96–1.15)
Depressive symptoms	413 (5.1)	2.98 (2.39–3.72)	3.04 (2.43–3.80)	2.10 (1.66–2.66)	2.10 (1.65–2.66)

OR: Odds ratios; CI: confidence interval; Lack of physical activity: people doing aerobic HEPA <150 min a week. Model 1: unadjusted; Model 2: age, education level, partnership status; Model 3: Model 2 + each of 16 chronic somatic illnesses, smoking status, alcohol intake, BMI; Model 4: Model 3 + depressive symptoms for lack of HEPA and lack of HEPA for depressive symptoms.

**Table 3:** Crude and adjusted ORs for inpatient health care utilisation related to physical inactivity and depressive symptoms.

Inpatient Health Care Utilisation					
	All *N* (%)	Model 1 (Unadjusted) OR (95% CI)	Model 2 OR (95% CI)	Model 3 OR (95% CI)	Model 4 OR (95% CI)
**Men**					
Lack of health enhancing physical activity	3598 (46.9)	1.07 (0.94–1.21)	0.98 (0.86–1.12)	0.90 (0.78–1.03)	0.89 (0.78–1.03)
Depressive symptoms	260 (3.4)	2.74 (2.09–3.60)	2.54 (1.91–3.37)	1.50 (1.09–2.08)	1.52 (1.09–2.10)
**Women**					
Lack of health enhancing physical activity	4276 (52.8)	1.32 (1.17–1.50)	1.24 (1.09–1.40)	1.08 (0.95–1.23)	1.06 (0.93–1.21)
Depressive symptoms	413 (5.1)	2.70 (2.16–3.34)	2.76 (2.20–3.44)	2.10 (1.64–2.69)	2.09 (1.64–2.68)

OR: Odds ratios; CI: confidence interval; Lack of physical activity: people doing aerobic HEPA <150 min a week. Model 1: unadjusted; Model 2: age, education level, partnership status; Model 3: Model 2 + each of 16 chronic somatic illnesses, smoking status, alcohol intake, BMI; Model 4: Model 3 + depressive symptoms for lack of HEPA and lack of HEPA for depressive symptoms.

**Table 4:** Combination of depressive symptoms and physical inactivity towards outpatient health care utilisation.

Lack of Health Enhancing Physical Activity	Depressive Symptoms	Health Care Utilisation, *N* (%)	Model 1 OR (95% CI)	Model 2 OR (95% CI)	Model 3 OR (95% CI)
**Men**					
+	+	108 (65.5)	2.89 (2.08–4.00)	2.28 (1.63–3.18)	1.43 (0.99–2.07)
-	+	62 (65.3)	2.92 (1.90–4.48)	2.73 (1.76–4.22)	1.51 (0.95–2.41)
+	-	1362 (39.7)	1.01 (0.92–1.11)	0.93 (0.85–1.03)	0.89 (0.80–0.98)
-	-	1571 (39.5)	1	1	1
Synergy index			0.98 (0.44–2.19)	0.77 (0.31–1.92)	0.19 (0.14–8.72)
Attributable proportion			−0.02 (−0.55–0.52)	−0.17 (−0.81–0.47)	0.02 (−0.57–0.62)
Relative excess risk due to interaction			−0.04 (−1.58–1.50)	−0.38 (−1.77–1.01)	0.03 (−0.82–0.89)
**Women**					
+	+	195 (73.0)	3.28 (2.48–4.33)	3.22 (2.43–4.26)	2.02 (1.50–2.73)
-	+	106 (72.1)	3.10 (2.14–4.46)	3.26 (2.26–4.72)	2.40 (1.64–3.52)
+	-	1960 (48.9)	1.15 (1.05–1.26)	1.12 (1.03–1.23)	1.06 (0.97–1.17)
-	-	1671 (45.4)	1	1	1
Synergy index			1.01 (0.35–2.98)	0.93 (0.94–1.76)	0.70 (0.31–1.60)
Attributable proportion			0.01 (−0.23–0.75)	−0.05 (−0.52–0.42)	−0.22 (0.78–0.34)
Relative excess risk due to interaction			0.03 (−2.39–2.45)	−0.17 (−1.64–1.31)	−0.44 (−1.51–0.63)

OR: Odds ratios; CI: confidence interval. Model 1: unadjusted; Model 2: age, education level, partnership status; Model 3: Model 2 + each of 16 chronic somatic illnesses, smoking status, alcohol intake, and body mass index.

**Table 5:** Combination of depressive symptoms and physical inactivity towards inpatient health care utilisation.

Lack of Health Enhancing Physical Activity	Depressive Symptoms	Health Care Utilisation, *N* (%)	Model 1 OR (95% CI)	Model 2 OR (95% CI)	Model 3 OR (95% CI)
**Men**					
+	+	55 (33.3)	3.16 (2.26–4.42)	2.64 (1.86–3.75)	1.52 (1.02–2.26)
-	+	24 (25.3)	2.13 (1.33–3.42)	2.14 (1.32–3.50)	1.19 (0.64–2.04)
+	-	473 (13.8)	1.01 (0.89–1.16)	0.94 (0.82–1.08)	0.88 (0.76–1.01)
-	-	542 (13.6)	1	1	1
Synergy index			0.45 (0.22–0.93)	1.51 (0.50–4.54)	7.57 (0.00–130,362)
Attributable proportion			−0.84 (−1.88–0.20)	0.21 (−0.26–0.68)	0.30 (−0.19–0.78)
Relative excess risk due to interaction			−2.65 (−5.54–0.25)	0.55 (−0.81–1.91)	0.45 (−0.39–1.28)
**Women**					
+	+	78 (29.2)	2.89 (2.18–3.83)	2.72 (2.04–3.63)	1.75 (1.28–2.40)
-	+	51 (34.7)	3.70 (2.60–5.27)	4.05 (2.82–5.82)	3.49 (2.40–5.01)
+	-	646 (16.1)	1.34 (1.18–1.53)	1.26 (1.11–1.44)	1.13 (0.99–1.30)
-	-	460 (12.5)	1	1	1
Synergy index			0.62 (0.35–1.11)	0.52 (0.29–0.95)	0.29 (0.13–0.65)
Attributable proportion			−0.40 (−0.98–0.18)	−0.59 (−1.26–0.09)	−1.07 (−2.00 to −0.14)
Relative excess risk due to interaction			−1.16 (−2.65–0.34)	−1.59 (−3.20–0.01)	−1.87 (−3.25 to −0.50)

OR: Odds ratios; CI: confidence interval. Model 1: unadjusted; Model 2: age, education level, partnership status; Model 3: Model 2 + each of 16 chronic somatic illnesses, smoking status, alcohol intake, and body mass index.
